# Processing Written Language in Video Games: An Eye-Tracking Study on Subtitled Instructions

**DOI:** 10.3390/jemr18050044

**Published:** 2025-09-17

**Authors:** Haiting Lan, Sixin Liao, Jan-Louis Kruger, Michael J. Richardson

**Affiliations:** 1 Department of Linguistics, Macquarie University, Wallumattagal Campus, Macquarie Park, NSW 2109, Australia; sixin.liao@mq.edu.au (S.L.); janlouis.kruger@mq.edu.au (J.-L.K.); 2 Department of Translation, Interpreting and Intercultural Studies, Hong Kong Baptist University, Kowloon, Hong Kong 999077, China; 3 UPSET Research Focus Area, North-West University, Vanderbijlpark 1900, South Africa; 4 Performance and Expertise Research Center, Macquarie University, Wallumattagal Campus, Macquarie Park, NSW 2109, Australia; michael.j.richardson@mq.edu.au; 5 School of Psychological Sciences, Macquarie University, Wallumattagal Campus, Macquarie Park, NSW 2109, Australia

**Keywords:** text processing, subtitle processing, eye movements, attentional allocation, video games

## Abstract

Written language is a common component among the multimodal representations that help players construct meanings and guide actions in video games. However, how players process texts in video games remains underexplored. To address this, the current exploratory eye-tracking study examines how players processed subtitled instructions and resultant game performance. Sixty-four participants were recruited to play a videogame set in a foggy desert, where they were guided by subtitled instructions to locate, corral, and contain robot agents (targets). These instructions were manipulated into three modalities: visual-only (with subtitled instructions only), auditory only (with spoken instructions), and visual–auditory (with both subtitled and spoken instructions). The instructions were addressed to participants (as relevant subtitles) or their AI teammates (as irrelevant subtitles). Subtitle-level results of eye movements showed that participants primarily focused on the relevant subtitles, as evidenced by more fixations and higher dwell time percentages. Moreover, the word-level results indicate that participants showed lower skipping rates, more fixations, and higher dwell time percentages on words loaded with immediate action-related information, especially in the absence of audio. No significant differences were found in player performance across conditions. The findings of this study contribute to a better understanding of subtitle processing in video games and, more broadly, text processing in multimedia contexts. Implications for future research on digital literacy and computer-mediated text processing are discussed.

## 1. Introduction

Digital media innovations are reshaping literacy practices and communicative norms [1]. A growing range of digital media now supports language learning, including mobile-assisted PC-based applications and video games [1,2,3,4]. Importantly, digital skills among individuals no longer fall neatly into the dichotomy of “digital natives” and “digital immigrants”; instead, there is large variability in how individuals consume media content [1]. The tremendous influence of digital media on both societies and individuals has stimulated extensive discussion about the relationship between digital literacies and technology-assisted learning in both academia and industry [1]. In other words, the discussion is fundamentally about how people engage with the content of digital media and how such digital engagement may vary. For example, in the context of language learning, understanding how readers process texts across different forms of digital media can help educators and media designers create platforms that are customized, efficient, and learning-friendly.

### 1.1. Subtitles in Video Games and Digital Literacy

Subtitles are one of the most prevalent texts for comprehension and learning in digital multimedia such as videos and video games. For example, many commercial games now include subtitled instructions or captions to help players learn the rules of the games. Previous research has shown that video games can provide effective platforms and communities for language learning [5,6,7,8]. For example, for second language learners, playing video games in foreign languages facilitates incidental language learning, while participation in gaming communities enhances motivation to improve their language proficiency [1,6]. In other digital media, subtitles represent a contemporary form of computer-mediated learning [9,10,11]. Although subtitles are pervasively present in various forms and flexible in digital media, previous research mainly focused on how people process subtitles cognitively in non-interactive media (i.e., media that is consumed passively, such as videos) [12,13,14,15,16]. Relatively little is known about how they process texts in interactive multimedia, particularly video games. The interactions between players and video games are assumed to impact text processing behaviour (e.g., subtitle reading). In non-interactive multimedia such as subtitled films, subtitles primarily support information conveyed by the soundtrack and on-screen visuals. The cognitive processes involve mutual modulation between subtitle processing and the simultaneous processing of visuals, sounds, and their inter-related mechanics [17,18]. In contrast, video games add another layer of complexity. Unlike passive viewing, players not only process and integrate subtitles with other sensory inputs, but also respond dynamically to the game environment, making decisions and updating their actions in real-time. For example, in some adventure video games like *Detroit: Become Human*, the menu-based subtitled dialogues between the characters often determine how the game progresses. The interactions among the basic elements of language (i.e., verbal communication), actions, and their interpretable relations construct the meaning during gameplay and immerse players into the gaming world [19]. It is the rich multimodal representations for immersive meaning-making and the immediate feedback on players’ performance that make video games good materials for literacy development and language learning, as well as cognitive skill training in education and therapy [5,7,8,20].

However, it is worth noting that the interactivity between players’ actions and the presented content could bring both benefits and challenges to players’ general information processing. On the positive side, interactivity with multimedia enables users to have greater autonomy, as they can access immediate external support (e.g., written languages, icons, dynamic images) to actively and dynamically construct mental models [21]. But, on the other hand, information from multiple sources, combined with manual actions under a specific time restriction, may increase their players’ cognitive load [22] and constrain their attention allocation. Moreover, since viewers develop nested goals in video games [23], the goal hierarchy impacts players’ text-processing behaviors, such as what texts and which parts of texts need to be processed. That said, the prevalence of subtitles and their potential for literacy development and language learning [9,10,24,25] opens up questions about how subtitles are processed and how they may impact players’ performance in video games.

### 1.2. Theoretical Frameworks in Multimodal Processing

Theories of multimedia information processing provide complementary perspectives on how individuals process texts in multimodal contexts. Dual-Coding Theory (DCT; [26]) proposes two distinct representational systems, verbal and non-verbal (imagery), which can activate each other in systematic ways to promote learning and performance. Building on this assumption and the multicomponent model of working memory [27,28], the Cognitive Theory of Multimedia Learning (CTML; [29,30]) specifies that auditory and visual information is processed through separate but capacity-limited channels, with learning effectiveness depending on how text–image materials engage sensory memory, working memory, and long-term memory. Taking a step forward, the Integrated Model of Text and Picture Comprehension (ITPC; [31]) provides a more fine-grained account of comprehension, describing three successive stages—surface, propositional, and mental model representations—through which text–image information is integrated. On the other hand, more recent frameworks like the Scene Perception and Event Comprehension Theory (SPECT; [32]) and Visual Narrative Grammar (VNG; [33,34]) focus on multimodal comprehension in sequential image contexts such as comics, films, and storyboards. SPECT emphasizes semantic processing, showing how perceptual and memory-driven mechanisms jointly construct event models in working memory while sustaining them in long-term memory. VNG, by contrast, stresses syntactic organization, proposing that image sequences follow a narrative grammar that structures meaning at the discourse level. SPECT and VNG together explain how semantic and structural mechanisms jointly underpin sequential image comprehension. Additionally, more closely related to reading in multimedia, the Multimodal Integrated Language (MIL) framework [35] provides a specific account of how cognitive systems for attention, memory, language processing, and oculomotor control are coordinated to support the reading of subtitles with auditory and visual inputs in videos. It assumes the sequential allocation of attention to language-related representations, but allows for concurrent processing of verbal and non-verbal inputs. Built on the E-Z Reader—a computational model of eye-movement control during static reading [36]—it assumes the sequential allocation of attention for text processing, as proposed by E-Z Reader, but extends the model by incorporating parallel processing of verbal and non-verbal information from visual and auditory inputs.

Regarding digital literacies, two representative frameworks related to reading in media in general and reading in gameplay are exemplified here. The Reading as Problem Solving (RESOLV) model [37,38] is based on the observation that digital reading facilitates problem-solving and decision-making for different goals within certain physical and social contexts. The two major factors affecting digital reading are (1) contexts and (2) the tasks embedded in the contexts. Specifically, contexts include the physical and social environments and readers’ traits (e.g., motivation, interest, and ability), which are relatively stable. While reading tasks are continuously updated and adapted to readers’ embedded contexts. Both contexts (e.g., different media) and the task sets determine readers’ decisions and actions on reading processes, strategies, and outcomes. Moreover, the model stresses that reading itself is dynamic due to the limited cognitive resources and constant balancing of benefits and costs [37].

On the other hand, the Gamer Response and Decision (GRAD) Framework [23,39] seeks to explain how individuals navigate and process various multimodal symbols to make decisions and learn within video games. These multimodal symbols can be broadly categorized into written language, oral language, visual representations, audio sources, and tactile experiences. Under this categorization, subtitles in video games are primarily written language. Specifically, the multimodal symbols constitute the player’s experience (e.g., dialogue in cutscenes) and assign affordances to the in-game elements (e.g., whether a block is movable or not), which impact how players translate their understanding into dynamic and nested goals [23]. The nested goals define the actions of the players. For example, the overarching goal of completing the game branches into sub-tasks, such as defeating enemies, which further divide into smaller, actionable tasks like how to eliminate an enemy [23]. Throughout the gaming experience, meta-cognition modulates the process of self-regulating, planning, evaluating, and monitoring actions [40].

Together, these multimodal theories or frameworks primarily explain how verbal, visual, and auditory information are represented and integrated during multimedia comprehension. They focus on the structural assumptions of multimodal information processing, including dual-channel representation, limited working memory capacity, and the sequential or concurrent allocation of attentional resources. Building on these accounts, the present study also considers higher-order executive functions that mediate players’ behaviour in interactive environments. While the aforementioned theories describe how multimodal inputs are encoded, integrated, and stored, executive functions [41,42], such as goal activation, monitoring, planning, shifting, and inhibition, help explain how individuals actively control and adapt their reading and action strategies in real-time. For example, players must set goals about whether to prioritise subtitles or visual cues, monitor their performance across modalities, plan action sequences, flexibly shift between reading and executing manual tasks, and inhibit irrelevant information to manage cognitive load. Together, multimodal processing theories and executive control functions offer a complementary perspective: the former outlines the representational and cognitive architecture of multimedia learning, while the latter describes the adaptive mechanisms through which players navigate multimodal inputs under interactive and time-pressured conditions.

This exploratory study aims to examine how players process subtitles in video games and how the subtitles impact players’ performance. To this end, we used a first-person desert herding videogame as the experimental platform, where we manipulated both the subtitle modality and subtitle relevancy within the game. The current exploratory study examines how the two variables affect players’ subtitle processing at the subtitle and word levels and their performance efficiency and outcomes. Investigating subtitle processing in video games provides insights into optimizing subtitling practices for different purposes in interactive multimedia (e.g., training and education), future empirical studies, and empirical evidence for theories related to reading in interactive multimedia.

We hypothesized that players would read subtitles in video games in a dynamic and context-dependent manner. In cognitively demanding situations, players would first need to decide whether to engage with written language at all, depending on available sources of information and their goals. If reading is necessary, they would quickly determine which texts are relevant and which parts should be prioritized. As the game progresses, they would continue to decide whether further reading is worthwhile and at what depth (careful reading, skimming, or keyword extraction). The resulting textual cues would then guide their in-game actions, with these steps evolving in a non-linear way. More specifically, because video games involve complex multimodal symbols and interactive mechanics that heavily tax cognitive resources, subtitles may often function less as full passages to be read and more as visual cues to conserve attention. Players may therefore focus on the identifiers and keywords most relevant to immediate tasks, especially when subtitles are predictable or repetitive, allocating attention in ways that involve skipping most of the text while relying on keywords and the visual first-person game state. Thus, we predicted that players may have longer and more fixations on relevant subtitles compared to irrelevant subtitles, and that they would focus more on keywords rather than processing entire subtitles.

## 2. Methods

The current study aims to investigate how players process subtitles and decision-making outcomes (i.e., performance) in video games by examining eye-movement patterns during gameplay. We required participants to play a multiplayer videogame with a team of AI agents. The objective of the herding game was for a team of three “ground players” (GPs) and an “operator” to search for and corral “target agents” (TAs) randomly distributed around a containment area. The participants played as one of the GPs, teaming up with two autonomous artificial intelligence (AI) agents. The human player controlled humanoid avatars from a first-person perspective. The human player was responsible for herding and containing the target agents, while the two AI ground players patrolled around. However, as the desert was very foggy with a limited visibility of 5 m, the players needed to rely on the instructions provided by an additional AI drone operator (with global observation) to know which direction to go and which target agent to herd. These instructions were presented in three modalities: visual-only (subtitles only), auditory only (spoken instructions), and visual–auditory (a combination of both).

### 2.1. Materials and Design

Participants played a first-person multiplayer videogame called Desert Herding, which was designed using the Unity3D Game Engine (Version 202.3 LTS; Unity Technologies, 2023), with similar parameters to Prants et al. [43] and Simpson, Stening, et al. [44]. The task was completed in sound-isolated booths, each fitted with a condenser microphone headset, over-ear headphones, a computer monitor, a standard keyboard, and a standard mouse. An EyeLink Duo Portable eye-tracker with a sampling rate of 1000 Hz was used to record participants’ eye movements during game playing. SR Research WebLink software was used to present the stimuli. The experimental materials were displayed using HP computers with Intel i7 processors, 16 GB of RAM, RTX3070 GPUs, and on 27-inch monitors.

The game took place in a 500 × 500 m undulating 3D desert terrain, bounded by mountains, with a blue translucent ring indicating the containment area (10 m wide) at the center of the desert. Simulated fog reduced visibility to about 5 m, making players rely on in-game instructions to complete the herding tasks. Players had to herd one assigned TA at a time, as directed by the instructions, into the containment area. The instructions were generated by a custom-trained large language model (which is specified below).

**Ground players (GPs):** The participant played a dominant role in herding, while two AI players patrolled the area. The participant was named “Alpha”, while the two AI players were named “Bravo” and “Charlie”. The game began with GPs positioned within 100 m of the containment area in a pre-designed order. Players controlled their avatars using the mouse to change orientation and the “W”, “A”, “S”, and “D” keys to move at 10 m/s. The player’s screen displayed their avatars’ names, compass heading, and remaining time. Each session lasted 7 min, and players had to follow a set layout sequence when capturing TAs to ensure comparability across conditions.

**Target agents (TAs):** The game started with 9 moving TAs, each marked with a number above their heads. The TAs, capsule-shaped robots, were randomly positioned within 180 m of the containment area at the start of the game. These robots accelerated away from GPs when approached (within 10 m), with speeds of up to 10 m/s, proportional to their distance from the GPs. When undisturbed, the TAs moved freely, displaying small amounts of Brownian motion at about 0.01 m/s. Their rings of colored lights provided feedback: orange when moving freely outside the containment area, red when fleeing a GP, and blue when moving freely inside the containment area. Every 30 s, a new TA spawned randomly at a different location in the desert. The spawning speed and trial length were based on insights from 12 pilot tests conducted during the game’s development.

**Subtitled instructions:** The subtitled instructions were issued with two computational components in the current experiment: a communication decision model and a communication generator. The first component governed when to give navigation instructions, what information should be provided, and to whom it should be directed. The second component conveyed this information to the GPs verbally.

- Communication Decision Model: Communication decisions were determined by comparing GP and TA locations and headings against a set of logic-based rules. These policies were derived from prior research, which investigated optimal movement dynamics and operator instructions in herding tasks [43,44,45,46].
- Communication Generator: Audio was recorded of each confederate reading text passages aloud for three minutes. The recordings were used to train voice clone models (ElevenLabs Inc., New York, U.S 2023), which were optimized to match each confederate’s gender and accent, producing near-indistinguishable vocal tonality. A large language model (LLM), GPT-4-0125-Preview (OpenAI, California, U.S., 2023), was used to generate the text-based instructions from the communication decision model output. “Function calls” were employed to restrict LLM instructions to those relevant to the task and to minimize LLM hallucinations. These constraints and the output of the LLM function calls were derived from previous research [44]. Finally, the assigned confederate’s voice clone model converted this text into speech.

To minimize variability, the LLM received the following fields (context, agent state) and produced constrained outputs in a fixed schema (action type, target index, and keyword allocation). Subtitles were identical across modalities and were generated from a fixed template that was pre-trained for the LLM. Target assignment order was kept identical for all participants within each modality, but differed between modalities. In other words, while participants experienced the same sequence of target assignments within a given modality, the sequence varied across modalities to prevent participants from anticipating targets based on previous conditions.

**Figure 1:**
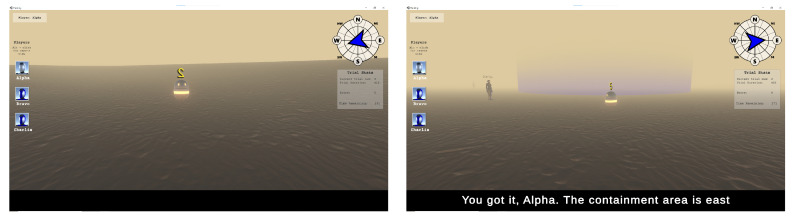
Screenshot of the gameplay in conditions with and without subtitles.

The subtitled instructions were displayed at 12 characters per second. Each subtitle could contain up to 63 characters in a line, and subtitles for each player were issued every five seconds. The time intervals for providing instructions to GPs were set to five seconds to ensure that each participant received an adequate number of instructions. The sentence structure included a comprehensive set of comprehensible command structures consisting of Name, Verb, Directional Words, Target Number, Destination, and Modifier. The subtitled instructions were presented at the bottom of the screen (see Figure 1). Each modality of the subtitled instructions (visual only, auditory only, or visual–auditory) lasted for 7 min, with the order of presentation randomized for each participant.

### 2.2. Participants

This was an exploratory study, so a formal sample size calculation was not carried out a priori. Sample sizes for subtitling studies conducted in the literature [18,47,48] have been undertaken with groups of 30–40 participants. Our sample comprised 64 subjects, substantially higher than previous studies. Further, while no significant differences were found in some instances, such as behavioral performance indicators (contained number, herding efficiency, containment efficiency), highly significant differences (ps < 0.001) were found in other aspects of the analysis, such as subtitle level analyses (fixation counts, dwell time percentages, but not subtitle skipping rates). These significant effects also indicate that the sample size is large enough to reveal some significant differences.

In total, 64 native English speakers (46 female, 18 male) aged from 18 to 29 (M = 20.17, SD = 2.3) from Macquarie University participated in this study in exchange for research course credits or AUD 25 gift vouchers. Participants identified themselves as non-dyslexic with normal or corrected-to-normal vision. The participants were required to have basic gaming experience with a keyboard and mouse. This study was approved by Macquarie University Human Research Ethics Committee (ethics approval number: 52024645755523). For details on the players’ gaming experience and subtitle experience, see Appendix A.

### 2.3. Procedure

Participants were tested individually in a soundproof lab. After entering the laboratory, participants reconfirmed whether they met the pre-screening conditions and provided written and verbal consent. Participants were seated 95 cm away from the screen and were asked to keep their overt eye movements within the focal range of the eye tracker. A 9-point calibration and eye-tracking validation process was then performed. Only eye-tracking calibrations that resulted in an average error value of less than 0.50 degrees and a maximum error value of less than 1.00 degrees were accepted to maximize tracking accuracy. Recalibration was performed until this criterion was met.

Participants then engaged in a 3 min practice trial, which mirrored the main experiment gaming trials, but without fog, allowing for them to understand the task setup and familiarize themselves with the game controls. Following this practice trial, participants completed the three experimental game trials, where visibility was reduced due to the presence of fog, requiring participants to rely on the in-game instructions to complete the task. Each modality of the subtitled instructions (visual only, auditory only, or visual–auditory) was presented for 7 min. The order of the three sessions was randomized for participants. After completing the experiment, participants filled out two questionnaires about their gaming habits and their experiences with subtitles in multimedia. The entire experiment took approximately 60–70 min.

### 2.4. Measures

**Figure 2:**
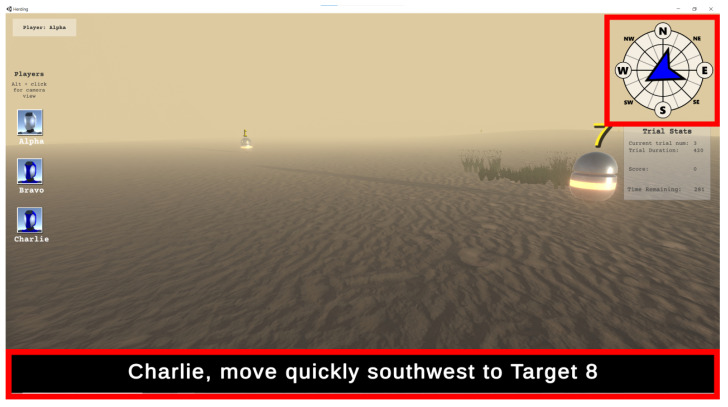
Two major interest areas for general attentional allocation (the red rectangles are the areas of interest).

To understand how participants processed subtitled instructions compared to other sources, we examined the participants’ overall attention allocation during gameplay by measuring how often they attended to the two key elements (areas of interest): the compass and the subtitle region (see Figure 2). Total fixation counts and dwell time on the two areas were compared. These eye-movement measures captured how much attention a participant gave to the subtitle region compared to the compass regions, where participants can obtain most of the directional information. And the directional information is the most directly related to their actions.

To understand how participants processed the subtitles at the subtitle level, we examined whether there were any differences in attentional allocation between subtitled instructions relevant to the player and those that were for the other ground players (i.e., Alpha vs. Bravo–Charlie). The eye-movement measures on each subtitle in each gaming modality include (1) total fixation counts (the total number of fixations in the subtitle region), (2) dwell time percentage (the proportion of total dwell time in the subtitle region to the duration of the subtitle), (3) subtitle skipping rates (the possibilities of the subtitles not being fixated), and (4) mean fixation duration (the mean fixation duration of the subtitles calculated by dividing total dwell time with total fixation counts).

To understand how participants processed the subtitles at the word level, the eye-movement data were used to explore whether there were any differences in attentional allocation to keywords within the subtitles. These keywords are high in informational value related to immediate actions in the current game. The keywords were selected from three categories: Names, Directional Words, and Target N (*N is the number assigned to each target agent).

The word-level eye-movement measures include (1) word skipping rates (the proportion of the words not being fixated, (2) fixation counts (the number of fixations of each word after being normalized by its occurrence and word length), and (3) mean fixation duration (the mean fixation duration of each word calculated by dividing dwell time by fixation counts). To reduce the impact of word length and word occurrence in this gaming context, the word-level data has been normalized by dividing the word-level data by the number of pixels that each word occupies on the screen and each word by the number of occurrences of that word. For example, the fixation count of each word is calculated as the total fixation count on each word divided by the total number of occurrences of the word and by the number of pixels occupied by the word.

To evaluate the effect of subtitle modalities on gameplay performance, three behavioral performance measures were used: (1) the number of target agents contained, (2) herding efficiency, and (3) containment efficiency. The number of target agents contained refers to the total number of robots captured during regular trials in each modality. Herding efficiency, calculated as D1/(T2 − T1), measures the efficiency of locating a moving target within the sensing range. Here, D1 is the distance traveled from the appearance of the instructional subtitle (T1) to the moment the target is located (T2), relative to the time elapsed (T2 − T1). Containment efficiency, calculated as D2/(T3 − T2), reflects the efficiency of driving the target into the containment area. D2 represents the distance traveled from locating the moving target (T2) to successfully driving it into the containment area (T3), relative to the time taken (T3 − T2). These performance metrics capture how participants navigated and completed tasks under different subtitle modalities.

### 2.5. Analysis

The compass AOI was defined as a rectangle with pixel coordinates [2170.0, 45.0, 2542.0, 411.0], and the subtitle AOI as a rectangle with coordinates [7.0, 1207.0, 2551.0, 1395.0]. When comparing dwell time and fixation counts, subtitle-level AOI area was not considered because subtitles were presented at a constant medium speed, which sufficiently allows for viewers to fully read them in video-based studies. At the word level, however, AOI size was taken into account, since word length varied; fixation measures were therefore normalized according to the pixel width of each word. Specifically, for word-level AOI, word-level bounding boxes were used to map fixations to individual words when subtitles were presented (as full sentences at once). The AOI size was defined such that the horizontal boundaries were set by equally dividing the pixel space between adjacent words, while the vertical boundaries extended 50 pixels above and below each word. This vertical margin was determined post hoc, based on the aggregated distribution of fixations within the subtitle area and any fixations outside this range were excluded.

Prior to model fitting, exploratory data analysis was conducted, including the use of histograms to inspect variable distributions and to identify potential transformations needed to improve model fit. The behavioral and eye-movement data were then analyzed using Generalized/Linear Mixed-Effects Models (G/LMMs) [49,50,51]. The models were built using the “lme4” [52] and “lmerTest” [53] packages in R (version 4.2.0), linear mixed models (LMMs) were fitted using restricted maximum likelihood (REML), whereas generalized linear mixed models (GLMMs) were fitted using maximum likelihood (ML). In this way, we can ensure unbiased estimates of variance components, thus improving the accuracy of fixed effects. The BOBYQA (Bound Optimization BY Quadratic Approximation) optimizer [54] was used to improve convergence, minimize data loss, and ensure accuracy in the large dataset [55].

For performance measures, the reference level for the modalities was set as the auditory-only modality in contrast to the visual–auditory and visual-only modalities. Modalities were treated as fixed effects, with participants as random effects. For subtitle-level eye -movement measures, the reference level was set as the visual-only modality in contrast to the visual–auditory modality, and irrelevant against relevant subtitles. Modalities, relevancy, and their interactions were treated as fixed effects, with participants as random effects. For word-level eye-movement measures, the reference level was set as the visual-only modality in contrast to the visual–auditory modality, and “Directional Words” against other word categories. Modalities, word categories, and their interactions were treated as fixed effects, with random effects for participants.

During model fitting, random slopes and intercepts were progressively removed from the maximal random effects structure to achieve the best fit with the data [52,56]. Additionally, all models were compared using ANOVA to identify the best-fitting model, followed by pairwise comparisons conducted using the “emmeans” package [57]. After fitting the models, diagnostic plots (e.g., Q-Q plots and residual vs. fitted plots) were used to check assumptions of normality, homoscedasticity, and independence of residuals, with necessary transformations applied to ensure that these assumptions were met. A total of 64 participants were recruited. Due to missing or incomplete data, the analyses included 62 participants for performance, 63 for word-level analysis, and 64 for subtitle-level analysis. For further details, see Appendix B.

## 3. Results

### 3.1. Behavioral Performance

*Contained number.* A generalized linear mixed model (GLMM) with a Poisson distribution was used to evaluate the effect of different modalities on the contained number of target agents. The model included a random intercept for participants to account for individual differences. The average number of target agents being contained varied considerably between participants. This is reflected in the GLMM results, where the Marginal $R^{2}$ was 0.006 and the Conditional $R^{2}$ was 0.285. Participants performed similarly (F (2, 173) = 0.684, *p* > 0.05) in auditory-only modality (M = 5.71, SD = 2.17), visual–auditory modality (M = 5.81, SD = 2.37), and visual-only modality (M = 6.20, SD = 2.52). Pairwise comparisons on the log scale showed no significant differences between conditions: auditory only vs. visual only (estimate = −0.088, 95% CI [−0.239, 0.062], *p* = 0.480), auditory only vs. visual–auditory (estimate = −0.033, 95% CI [−0.185, 0.119], *p* = 0.907), and visual only vs. visual–auditory (estimate = 0.056, 95% CI [−0.090, 0.202], *p* = 0.731).

*Herding efficiency.* A generalized linear mixed model (GLMM) with a Gamma distribution and log link was used to evaluate the effect of different modalities on herding efficiency. The model included a random intercept for participants to account for individual differences. The herding efficiency again varied considerably between participants. This is reflected in the LMM results, where the marginal $R^{2}$ was 0.011 and the conditional $R^{2}$ was 0.144. Participants performed similarly ($\chi^{2}\left(2\right)$ = 1.75, *p* = 0.417) in visual–auditory modality (M = 166.71, SD = 124.24), auditory-only modality (M = 180.50, SD = 117.39), and visual-only modality (M = 197.96, SD = 133.84). Pairwise comparisons on the log scale confirmed no significant differences between conditions: auditory only vs. visual only (estimate = −0.088, 95% CI [−0.363, 0.187], *p* = 0.806), auditory only vs. visual–auditory (estimate = 0.094, 95% CI [−0.177, 0.364], *p* = 0.776), and visual only vs. visual–auditory (estimate = 0.181, 95% CI [−0.085, 0.447], *p* = 0.380).

*Containment efficiency.* A generalized linear mixed model (GLMM) with a Gamma distribution and log link was used to evaluate the effect of different modalities on containment efficiency. The model included a random intercept for participants to account for individual differences. Like in the other two performance measures, the containment efficiency varied considerably between participants. This is reflected in the LMM results, where the marginal $R^{2}$ was 0.026 and the conditional $R^{2}$ was 0.130. Participants performed similarly ($\chi^{2}\left(2\right)$ = 4.51, *p* = 0.105) in the visual–auditory modality (M = 445.27, SD = 236.22), visual-only modality (M = 458.68, SD = 240.55), and auditory-only modality (M = 534.55, SD = 240.82). Pairwise comparisons on the log scale indicated no significant differences between conditions: auditory only vs. visual only (estimate = 0.161, 95% CI [−0.027, 0.349], *p* = 0.216), auditory only vs. visual–auditory (estimate = 0.189, 95% CI [−0.000, 0.378], *p* = 0.116), and visual only vs. visual–auditory (estimate = 0.028, 95% CI [−0.156, 0.212], *p* = 0.951).

### 3.2. Visual Attention Allocation on Directional Information

**Figure 3:**
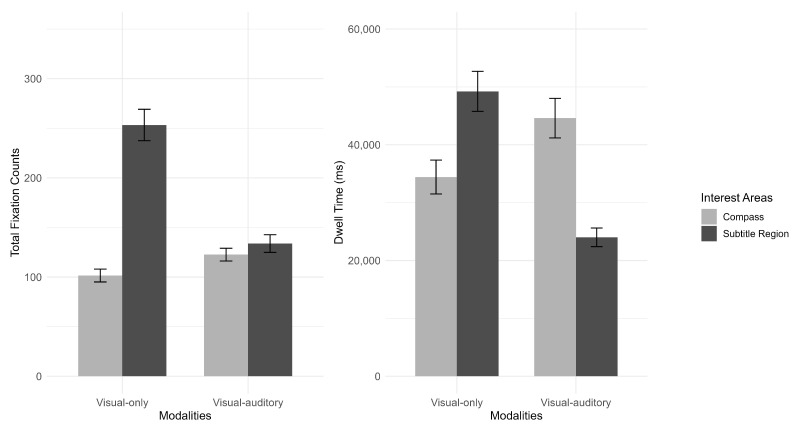
Total fixation counts and dwell time on compass and subtitle region in visual-only and visual–auditory modality (The error bars represented standard error).

In the game, participants obtained directional information from the compass, the subtitles, and/or the audio to finish the tasks. When comparing the two modalities with subtitles (visual only vs. visual–auditory), participants paid more attention to the subtitles in the visual-only modality, as indicated by longer dwell time and more total fixation counts on the subtitle region (Figure 3). In the visual–auditory modality, participants shifted more attention to the compass, as shown by more fixation counts and longer dwell time on the compass compared to the pattern in the visual-only modality. In the visual–auditory modality, participants used both the subtitles and the soundtrack as a dual-channel approach to receiving instructions, with both channels working simultaneously and complementing each other.

### 3.3. Subtitle-Level Analysis: Modality and Relevancy

**Figure 4:**
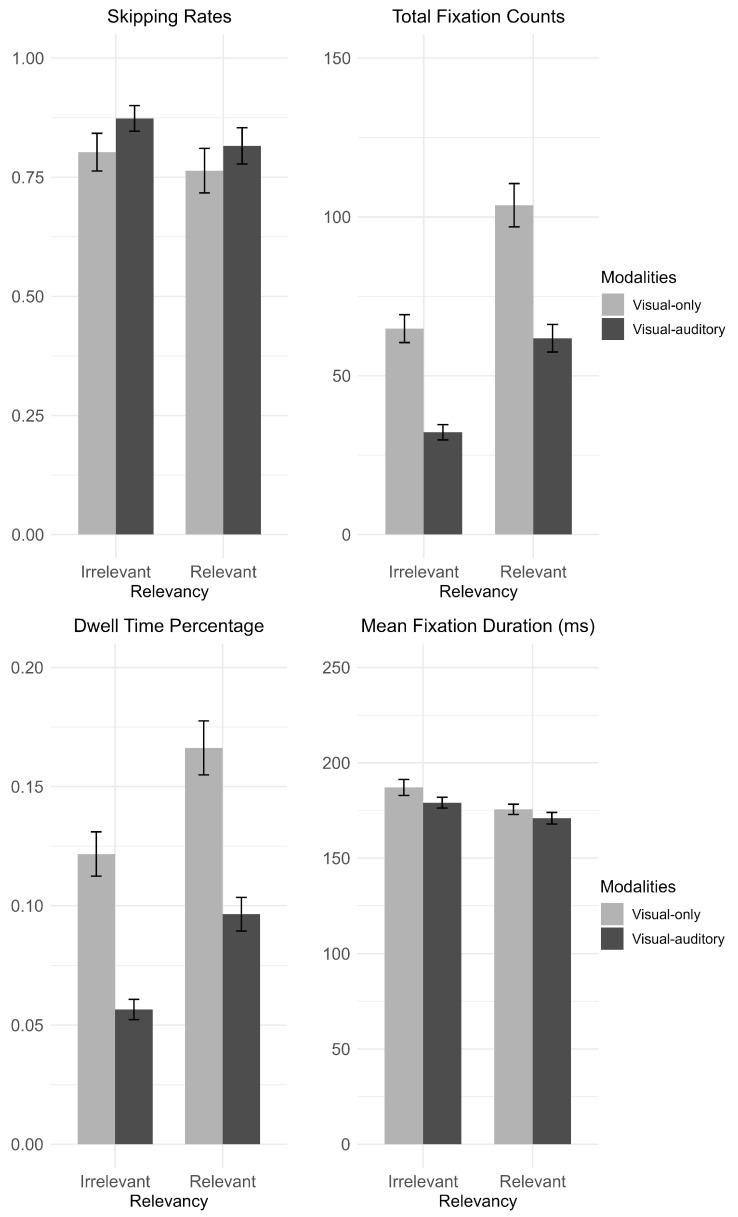
Modality-level eye-movement measures (The error bars represented standard error).

The subtitle-level analysis focuses on how subtitle modalities and relevancy affect the processing of all subtitles. The modalities analyzed here are visual-only and visual–auditory modalities. The relevancy of the subtitles is defined as whether the subtitles were issued to the participants (“relevant”) or others (“irrelevant”). Being able to distinguish the relevancy of subtitles is a sign of subtitle processing in gaming contexts. Item-level random effects (e.g., for individual subtitles or words) were not included because several measures were aggregated at the session level, and thus item-level information was no longer available. In addition, we compared alternative model specifications using ANOVA and found that the model with participant random intercepts provided the best fit. The mean and standard deviation and G/LMMs results for each text-level measure are shown in Figure 4.

*Total fixation counts:* The total fixation counts were calculated as the total number of fixations on subtitles aggregated by each modality. As the fixation counts were aggregated by each modality session, the total fixation counts were first subjected to a square root transformation after considering its distribution before being fitted to a linear mixed model. Main effects were found for subtitle modality (estimate = −2.135, 95% CI [−2.647, −1.623], *p* < 0.001) and subtitle relevancy (estimate = −2.033, 95% CI [−2.474, −1.592], *p* < 0.001). No interaction effect was found (estimate = −0.106, 95% CI [−0.732, 0.521], *p* = 0.741). These results indicate that participants fixated significantly more on the subtitles in the visual-only modality than in the visual–auditory modality, and significantly more on the relevant subtitles than on the irrelevant subtitles.

*Dwell time percentage:* The dwell time percentage was calculated as the dwell time on each subtitle divided by the presentation time of the subtitle aggregated by each modality. As the dwell time percentage was aggregated by each modality session, the dwell time percentage was first subjected to a square root transformation after considering its distribution before being fitted to a linear mixed model. Main effects were found for subtitle modality (estimate = −0.089, 95% CI [−0.111, −0.068], *p* < 0.001) and subtitle relevancy (estimate = −0.058, 95% CI [−0.077, −0.040], *p* < 0.001). No interaction effect was found (estimate = −0.013, 95% CI [−0.040, 0.014], *p* = 0.354). These results indicate that participants fixated significantly longer on subtitles in the visual-only modality than in the visual–auditory modality, and significantly longer on the relevant subtitles than on the irrelevant subtitles.

*Subtitle skipping rates:* Subtitle skipping rates were calculated as the number of subtitles that were not fixated at least once divided by the total number of subtitles during each modality. In other words, subtitle skipping rates are calculated as the probability of each subtitle being skipped in each modality. The subtitle skipping rates were analyzed using a generalized linear mixed model with a binomial distribution. No significant main effects were found for subtitle modality (estimate = 0.428, 95% CI [−0.484, 1.340], *p* = 0.358) or subtitle relevancy (estimate = 0.205, 95% CI [−0.559, 0.969], *p* = 0.600). No significant interaction effect was found (estimate = 0.895, 95% CI [−0.321, 2.111], *p* = 0.149). These results indicate that participants skipped subtitles to a similar extent across modalities and relevancy.

*Mean fixation duration:* Mean fixation duration was calculated by dividing the dwell time by the total number of fixations on each subtitle aggregated by each modality. As the mean fixation duration was aggregated by each modality session, the mean fixation duration was first subjected to a square root transformation after considering its distribution before being fitted to a linear mixed model. No significant main effect was found for subtitle modality (estimate = −0.159, 95% CI [−0.422, 0.104], *p* = 0.237). A significant main effect of subtitle relevancy was found (estimate = 0.387, 95% CI [0.158, 0.615], *p* = 0.001). No significant interaction effect was found (estimate = −0.108, 95% CI [−0.431, 0.214], *p* = 0.510). These results indicate that the mean fixation duration on irrelevant subtitles was longer than that on relevant subtitles.

As a sanity check, essential instructions (i.e., when a new target agent is assigned) were isolated and analyzed, as shown in Appendix C. The overall results are consistent with the current subtitle-level analysis.

### 3.4. Word-Level Analysis: Modality and Word Categories

**Figure 5:**
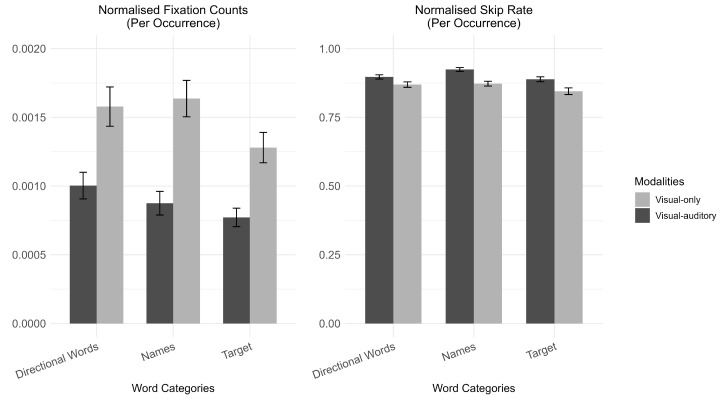
Word-level eye-movement measures (the error bars represented standard error).

The more direct evidence of word processing in subtitles is whether participants can prioritize word categories for their immediate actions in the relevant subtitles. For the word-level analysis in this section, we compared eye-movement measures on three-word categories in visual-only and visual–auditory modalities (Figure 5). As mentioned, the three-word categories were selected based on their relevancy to immediate actions in descending order: Directional Words, Names, and the number of the target agent (“Target N”).

Apart from skipping rates, only the data of the fixated words were analyzed. This was because we were primarily interested in how words were processed foveally and avoided the ambiguity of whether words were not processed or parafoveally processed. Moreover, the eye-movement data have been normalized based on word length and occurrence. The word length is calculated as the number of pixels that each word occupies on the screen.

*Word skipping rate:* The raw data for the word skipping rate of each word is standardized as word skipping rate = (total skipped occurrences/total occurrences). The word skipping rates were analyzed using a generalized linear mixed model with a binomial distribution. Participants exhibited a significantly higher word skipping rate in the visual–auditory modality compared to the visual-only modality (estimate = 0.279, 95% CI [0.158, 0.400], *p* < 0.001). Interactions were found where *p*-values were adjusted with the Tukey method. In particular, “Names” were skipped more frequently in the visual–auditory modality compared to the visual-only modality (estimate = −0.590, 95% CI [−0.721, −0.459], *p* < 0.001), a pattern not consistently observed in other word categories or modality combinations (see Table 1 and Table 2).

*Fixation counts.* The raw data for the fixation count of each word is standardized as the fixation count of each word = [(total fixation counts/occurrence)/number of pixels]. As the fixation counts on each word were standardized as continuous positive values, the fixation counts were analyzed with a linear mixed model after being log-transformed. Participants had more fixations in the visual-only modality than in the visual–auditory modality (estimate = 0.224, 95% CI [0.076, 0.372], *p* = 0.003). In the simple effect analysis, degrees of freedom were adjusted with the Kenward–Roger method and *p*-values were adjusted with the Tukey method. Interaction effects were found, where “Names” (estimate = 0.649, 95% CI [0.366, 0.932], *p* < 0.001) and “Target N” (estimate = 0.562, 95% CI [0.283, 0.841], *p* = 0.001) were more likely to be fixated in the visual-only modality than in the visual–auditory modality, as shown in Table 1 and Table 2.

*Mean fixation duration:* The raw data for the mean fixation duration of each word is standardized as mean fixation duration = [(total fixation duration/total fixation counts)/number of pixels]. As the mean fixation duration on each word was standardized as continuous positive values, the fixation counts were analyzed with a linear mixed model after being log-transformed. No significant effects were found for subtitle modality (estimate = −0.044, 95% CI [−0.089, 0.001], *p* = 0.055), word category “Names” (estimate = −0.033, 95% CI [−0.099, 0.033], *p* = 0.340) and “Target N” (estimate = −0.018, 95% CI [−0.085, 0.048], *p* = 0.593), or their interactions (ps > 0.05).

## 4. Discussion

This study explores how players process subtitles in video games and how the subtitle processing may impact their gaming performance. The findings indicate that, at the subtitle level, participants were able to evaluate and monitor their need for the additional support provided by subtitles. This was evidenced by more fixations and a higher dwell time percentage for subtitles relevant to the task (compared to irrelevant ones). Participants also focused more on subtitles when the audio was absent. At the word level, participants evaluated and monitored the importance of specific textual information in the subtitles (e.g., keywords) based on their task goals and immediate actions, and whether audio was available. This was particularly evident in their decreased attention to less consistently valuable information about actions (i.e., Names) when the audio was present. In other words, when audio was present, players tended to offload their visual demands by processing relatively lower informational value (e.g., Names) in the auditory channel, and to lessen their attention and physical demands. Moreover, such subtitle- and word-level processing strategies did not affect the efficiency and outcome of players’ gaming performance across conditions. The findings from this exploratory study provide initial insights into how people process written language in highly engaging and interactive multimedia (i.e., video games), laying a foundation for future empirical studies with similar research interests.

### 4.1. Gaming Performance and General Processing

For gaming performance, no significant differences were found in performance efficiency (herding efficiency and containment efficiency) and outcomes (contained number) across the three modalities. That said, the similar performance across conditions did not reflect how exactly players process subtitles in the current game, which suggests the importance of introducing eye-movement measures to gauge the role of text processing in the gaming contexts.

In the current game, the compass and subtitle regions are the primary sources of information for the upcoming actions. The availability of the subtitles and soundtrack affected participants’ attention to the compass and subtitle region. Under the visual-only condition (where audio was absent), participants paid more attention to the subtitle region, with more and longer fixations than on the compass or the subtitle region under the visual–auditory condition (where both subtitles and audio were present). The factors influencing this attention allocation pattern can be the dynamically shifting priorities and the completeness of information throughout the gameplay. Overall, the compass-subtitle attention allocation pattern in the current study is consistent with the task model proposed by the RESOLV model and GRAD framework, where processing multimodal representations is dynamically adjusted based on both the general contexts and constantly changing tasks [23,37].

### 4.2. Subtitle-Level Processing

For the subtitle-level processing, participants strategically processed subtitles by first identifying and attending to relevant subtitles. Compared to irrelevant subtitles, they exhibited more fixation counts and higher dwell time percentages on relevant subtitles. This pattern is consistent with findings from reading studies, where participants allocate more attention to content relevant to their reading goals [58]. The rationale for neglecting the irrelevant subtitles could be to save more visual resources for dominant information sources in video games–images.

Participants also processed subtitles by relying on the modality. In the visual–auditory modality, subtitles primarily complemented auditory input. Compared to the visual-only modality, participants exhibited fewer fixations and lower dwell time percentages on subtitles in the visual–auditory modality, as they were able to gather information from both channels. Moreover, even in the visual–auditory modality, subtitles are still useful in helping players to retain information. This is because subtitles presented entire sentences at once and remained on screen for a period, providing a stable reference to fill gaps in auditory attention caused by the transient nature of spoken words. This strategic use of subtitles was also reflected in participant feedback: “When the subtitle sentence was shown to me in full, it helped in case I wasn’t paying attention (to hearing), and I could look down and see the (target) number”.

Meanwhile, the high skipping rates in the current study raised intriguing questions on parafoveal processing. Studies on subtitles in non-interactive multimedia (i.e., videos) have shown that the word skipping rate in subtitles increases with speed. According to Kruger et al. [17], when subtitle speeds were 12 cps, 20 cps, and 28 cps, the word skipping rates were 29%, 35%, and 43%, respectively. In our experiment, participants exhibited a subtitle skipping rate of around 73% to 87% when reading subtitles in the current videogame, depending on the modalities, relevancy, and necessity of the subtitle. The highly cognitively demanding feature of video games can partly explain why players skipped subtitles to allocate attention to other sources of information. However, in the visual-only modality, participants had to rely entirely on subtitled instructions for movement information. Yet, despite this dependency, subtitle skipping rates remained above 76%. This prompts the following question: How were participants obtaining the necessary information if the skipping rates were so high?

Post-experiment feedback offers some insights. As one participant noted, “If I can hear the audio, I don’t really need to check, but I’ll notice it in my peripheral vision as confirmation of what I’ve heard”. This suggests that participants could process subtitles parafoveally or peripherally to gather supplementary information. This aligns with findings by Rayner [59], who observed that eye movements vary across different media. For example, in scene perception, fixation duration and saccade lengths are typically longer than in reading, influenced by both the task and the scene. Additionally, viewers can grasp the gist of a scene in as little as 40 ms [59]. While these insights hint at the mechanisms participants used to process subtitles in this gaming context, the exact extent of their attention span and reliance on parafoveal cues remains unclear. Future research could further explore these dynamics to deepen our understanding.

Moreover, the high subtitle and word skipping rates, selective attention to action-critical terms, and dual-channel coordination suggest that players strategically extract cues. As discussed in the theoretical frameworks introduced in the Introduction, humans naturally coordinate across modalities in daily comprehension and learning and selectively attend to salient or useful information. The results of the present study are consistent with previous findings that viewers strategically engage in computer-mediated environments. For example, during online searches, viewers often skim for salient and useful texts [60,61,62]. Eye-tracking studies have shown that, when readers skim webpages, they read more rapidly and use texts with hyperlinks as markers that facilitate efficient information extraction [60,63]. Another eye-tracking study further identified that viewers are guided by distinct signals when examining webpage information, where users transit from skimming to reading and from reading to possible clicking [61].

Finally, including participants as a random factor at the subtitle and word level greatly improved all models’ explanatory power (see Appendix B), emphasizing the role of individual differences in gaming. This variability is supported by questionnaire data showing that participants had an average of 7.81 years of gaming experience (SD = 4.77) and varied widely in the types of games they typically played (see Appendix A). The great individual variability in subtitle-level and word-level processing has several implications. First, this finding is consistent with the RESOLV model, which posits that individual traits (i.e., gaming experience or skill levels) could contribute to the observed differences in how people read in different media. Based on the current exploratory study, future studies could further investigate how variables like gameplay habits, cognitive abilities, and individual skill levels influence gaming performance and subtitle processing. But, more importantly, it aligns with the observation that diverse patterns of digital media use have shaped readers’ behavior to varying degrees [1]. Additionally, formal and informal media usage may interpenetrate, shaping the considerable variability in reading behaviors within video games. Consequently, age may not be a reliable indicator for categorizing digital literacy groups, echoing the debate over the distinction between “digital natives” and “digital immigrants” [1]. In this context, participants of similar age also vary considerably in processing texts during gameplay.

### 4.3. Word-Level Processing

In previous research on reading static texts, it was found that the word skipping rate is approximately 30%, with most skipped words being frequent, short functional words [59]. In the current study, the word skipping rates range from 84% to 92%, depending on the word categories and the modalities. The high word skipping rates suggested that players adopted a strategy of extracting textual cues sporadically rather than processing every word in the current context and task demands, which was also reflected in other eye-movement measures.

This sporadic processing is modulated by three factors. First are the modalities, as shown by higher word skipping rates, fewer fixation counts and lower dwell time percentage on keywords when the soundtrack was available (compared to when the soundtrack was absent). In the visual-only modality, participants depend entirely on the subtitles for instructions. In contrast, the visual–auditory modality provides more interrelated multisensory information, reducing the need for subtitles. The more participants can rely on auditory and visual channels in tandem, the less they depend on subtitles. Second, the features of the gaming environment play a role. The desert setting is a fixed, enclosed space, with the containment area consistently located at its center. The experienced players in the current study, especially those familiar with map-based games, can quickly internalize the layout of the desert area. This spatial familiarity further reduces their reliance on subtitles, contributing to the high subtitle-level and word-level skipping rates observed across modalities. Lastly, participants were required to constantly herd the moving targets to finish the gaming tasks in the current setting. Such high attention and physical demand incentivize participants to extract keywords sporadically to save more cognitive resources for processing imagistic information and manual movements.

Moreover, the interaction between modalities (visual–auditory modality) and word categories (i.e., Names) suggests that such sporadic word extraction was not random, but strategic, echoing the subtitle-level results. Such strategic processing is primarily reflected in how participants prioritize the three categories. As mentioned, subtitled instructions were given to GPs every five seconds to ensure each participant received an adequate amount, which means that Names and Target N were more predictable and stable than Directional Words. Thus, the three most important textual cues in descending order for participants to satisfy the immediate action needs were Directional Words, Target N, and Names from the logic of the game. The assumed priority of the information is found in the word-level analysis, where more prior information showed more standardized fixation counts and lower word skipping rates, as shown in interaction effects and the differences in slopes of the generalized linear mixed models or linear mixed models in Table 1 and Table 2.

Such processing prioritization of words was further influenced by the availability of supportive audio, as indicated by the interaction between modality and word categories. This was especially noticeable in the visual-only modality, where participants had to pay more attention to less immediate information for actions to avoid missing information, such as Names and Target N. In contrast, when audio was available, participants tended to offload their visual demands by processing words in the auditory channel, especially the less immediate action-related ones (i.e., Names). Overall, participants maintained relatively stable attention on Directional Words that were more critical for meeting immediate action demands compared to other words in both modalities. In sum, both the prioritization of word categories and the interaction between modality and word categories suggest that participants’ word extraction in the current game was strategic.

## 5. Implications for Future Research in Digital Literacy

By exploring text processing in a multimodal and interactive context, this study has implications for future research in digital literacy that focuses on how people engage with digital texts in various formats. For example, the substantial inter-individual differences observed in this study suggest that digital literacy (i.e., the proficiency of engaging with digital texts) may be shaped by various factors associated with the individuals (e.g., access to and experience with digital content), rather than by uniform competence within an age cohort. This finding supports the view that digital literacies are highly individualized, emphasizing the need to design customized experiences and foster diverse digital media skills.

In terms of practice, the results of this exploratory study offer concrete design guidance for digital literacy learning environments. Subtitled instructions, for instance, can serve as computer-mediated scaffolds for literacy development. Within learning management systems (LMS) or game-based activities, subtitles that highlight key action terms (e.g., directional words, targets), remain visible long enough for reinspection, and provide clear relevancy cues can effectively support learners’ strategic skimming under cognitive load. Features such as pace control (e.g., adjustable characters-per-second and dwell time) and modality choice (text-only versus audio–text) can help to calibrate cognitive demands. Furthermore, teacher-in-the-loop approaches that integrate caption-rich interactive tasks into institutional platforms (e.g., Moodle) and incorporate game-based mechanics may cultivate multiliteracies, such as rapid keyword extraction, cross-modal alignment, selective attention, and managing time-pressured instructions [62]. Echoing [1], this study argues for harnessing the transformation of literacy practices through digital media.

Future research could extend these insights by investigating how subtitle processing transfers to mobile-assisted language learning, telecollaboration, or LMS-integrated tasks, by comparing synchronous, time-pressured instructions with asynchronous captioned materials, and by modeling how factors such as motivation, autonomy, and prior platform use influence processing strategies and learning outcomes. This also underscores the importance of collecting direct measures of digital literacy and online learning experience, rather than inferring competence solely from age or gaming exposure.

## 6. Limitations

One limitation of this study lies in the controlled nature of the experimental environment, which was designed to minimize variables and ensure consistency. As a result, the subtitles’ functionality in our experiment was relatively simplistic compared to the more diverse textual formats found in commercial games. Future research could explore the effects of these more complex and varied text types on player experience and comprehension.

Additionally, concurrent subtitle presentation and manual actions may have imposed a substantial cognitive load on participants, potentially leading to higher subtitle and word skipping rates. To address this, future experiments could investigate the impact of varying time-lapse delays between actions and reading tasks, which may provide insights into participants’ text-processing dynamics under different cognitive conditions.

## 7. Conclusions

Changing contexts continue to modulate how written language is presented across media and how we engage with texts. The current exploratory study uses a self-developed video game to investigate how subtitle modalities and relevancy affect how players process texts in the current context. Arguably, the strongest contribution of this study is demonstrating what specific strategies that players used to extract information from texts in such a highly interactive multimodal context. At the macro level, players used multisensory information from different channels flexibly and in a coordinated fashion to finish the tasks. At the micro level, players distinguished the relevancy and necessity of the subtitles and the information values of different word categories across modalities. In our gaming context, texts were more likely to serve as textual cues that were extracted sporadically as needed. Moreover, we also found that inter-individual impacts yielded a stronger influence on subtitle processing in gameplay. No differences were found in gaming efficiency and outcomes.

The current exploratory study provides a possible method to uncover text processing strategies in interactive multimedia and tests theories related to text processing in multimedia. For practical implications, gaining insights into subtitle processing within interactive multimedia, such as video games, provides empirical insights into subtitling practices in language learning. For digital literacy, such contexts have the potential to test participants’ cognitive skills in extracting key information.

## Figures and Tables

**Table 1:** Pairwise comparison of word skipping and fixation counts by word categories.

Eye-Movement Measures	Category	Contrast	Estimate	SE	z/t Ratio	*p*-Value
Word Skipping Rates	Directional Words	Visual only/Visual–auditory	−0.28	0.06	−4.52	<0.0001
	Names	Visual only/Visual–auditory	−0.59	0.07	−8.81	<0.0001
	Target N	Visual only/Visual–auditory	−0.39	0.07	−5.70	<0.0001
Fixation Counts	Directional Words	Visual only/Visual–auditory	0.22	0.08	2.97	0.00
	Names	Visual only/Visual–auditory	0.65	0.14	4.49	<0.0001
	Target N	Visual only/Visual–auditory	0.56	0.14	3.96	0.00

**Table 2:** Pairwise comparison of word skipping rates and fixation counts by modalities.

Measure	Modality	Contrast	Estimate	SE	z/t Ratio	*p*-Value
Word Skipping Rates	Visual only modality	Directional Words–Names	−0.03	0.06	−0.53	0.59
		Directional Words–Target N	0.20	0.06	3.31	0.00
		Names–Target N	0.23	0.06	3.81	0.00
	Visual–auditory modality	Directional Words–Names	−0.34	0.07	−4.92	<0.0001
		Directional Words–Target N	0.09	0.07	1.37	0.17
		Names–Target N	0.44	0.07	5.98	<0.0001
Fixation Counts	Visual only modality	Directional Words–Names	−0.06	0.12	−0.51	0.61
		Directional Words–Target N	0.24	0.11	2.11	0.04
		Names–Target N	0.30	0.14	2.08	0.04
	Visual–auditory modality	Directional Words–Names	0.37	0.12	3.15	0.00
		Directional Words–Target N	0.58	0.11	5.07	<0.0001
		Names–Target N	0.21	0.14	1.49	0.14

**Figure A1:**
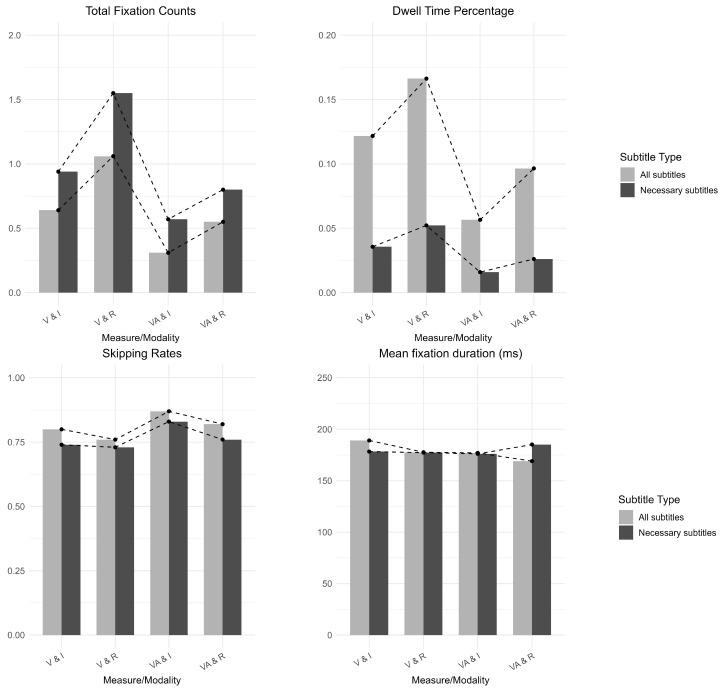
Essential subtitle-level eye-movement measures. V: visual-only modality; VA: visual–auditory modality; R: relevant subtitle; I: irrelevant subtitle.

## Data Availability

The data that support the findings of this study are available from the corresponding author upon reasonable request.

## Appendix A. Player Profiles: Gaming and Subtitle Experience

### Appendix A.1. Gaming Experience

Among the valid responses collected (N = 59), participants had been playing games for an average of 7.81 years (SD = 4.77). The types of games they played included cooperative games (N = 32), single-player games (N = 22), and no specific types (N = 5). When asked specifically about the genres of games they played (this was a multiple-choice option), 40 participants played action games, 35 played adventure games, 18 played role-playing games, and 31 played simulation games.

Participants reported various frequencies of gameplay, with twelve playing less frequently, ten playing daily, ten playing 4–5 times per week, ten playing once a week, nine playing monthly, seven playing 2–3 times per week, and one participant selecting “Other” and specifying a different frequency. Regarding the typical duration of each gaming session, seven participants played for half an hour, eight participants played for one hour, twenty-two participants played for an hour and a half, and twenty-one participants played for more than two hours. Regarding preferred control devices, the majority (39 participants) preferred using a keyboard and mouse, while 13 preferred joysticks, and 7 selected other types of devices.

### Appendix A.2. Subtitle Experience

Participants were asked a series of questions to evaluate their familiarity with subtitles. Among the valid responses collected (N = 59), when asked about their use of subtitles while watching content on video platforms (rated from 0 = Never to 10 = Always), the average score was 7.58 (SD = 2.88). In comparison, the use of subtitles during videogame gameplay (rated on the same scale) had a lower average of 4.43 (SD = 3.60).

Participants were also asked to rate the extent to which they found subtitles or other on-screen text distracting whilst playing video games, on a scale from 1 (Not at all) to 7 (Extremely), with an average score of 2.35 (SD = 1.67). Specifically, in this experiment, the level of distraction (scale of 1–7) caused by subtitles was rated with an average score of 1.90 (SD = 1.42). Conversely, when asked how helpful they found the subtitles during gameplay (scale of 1–7), participants gave an average score of 4.91 (SD = 1.78).

## Appendix B. Summary of G/LMM Models and Major Impact Factors

**Table A1:** Summary of G/LMM models and major impact factors.

Dependent Variables	Final Models	Summary of Major Factors Significantly Impact the Dependent Variables (*p* < 0.05)	Reference Level	Observations	Marginal/ Conditional $R^{2}$
Contained number	Modalities + (1|Subjects)	None	Modality: Auditory only	177	0.006/0.285
Herding efficiency	Modalities + (1|Subjects)	None	Modality: Auditory only	177	0.011/0.144
Containment efficiency	Modalities + (1|Subjects)	None	Modality: Auditory only	177	0.026/0.130
**Subtitle-level processing**					
Total Fixation Counts	Modalities ∗ Relevancy + (1|Subjects)	Visual–auditory (p<0.001); Irrelevant (p<0.001)	(1) Modality: Visual only(2) Relevancy: Relevant	384	0.250/0.751
Dwell Time Percentage	Modalities ∗ Relevancy + (1|Subjects)	Visual–auditory (p<0.001); Irrelevant (p<0.001)	(1) Modality: Visual only(2) Relevancy: Relevant	384	0.213/0.741
Subtitle Skipping Rate	Modalities ∗ Relevancy + (1|Subjects)	None	(1) Modality: Visual only(2) Relevancy: Relevant	384	0.087/0.290
Mean Fixation Duration	Modalities ∗ Relevancy + (1|Subjects)	Irrelevant (p=0.004)	(1) Modality: Visual only(2) Relevancy: Relevant	375	0.031/0.545
**Word-level processing**					
Word Skipping Rate	Modalities ∗ Word Category + (1|Subjects)	Visual-auditory (p<0.001); for interaction effects see Table 1 and Table 2	(1) Modality: Visual only(2) Word categories: Directional Words(3) Modality ∗ Word categories: Directional Words ∗ Visual only	29,118	0.017/0.165
Fixation Counts	Modalities ∗ Word Category + (1|Subjects)	Visual–auditory (p=0.003); for interaction effects, see Table 1 and Table 2	(1) Modality: Visual only(2) Word categories: Directional Words(3) Modality ∗ Word categories: Directional Words ∗ Visual only	648	0.072/0.364
Mean Fixation Duration	Modalities ∗ Word Category + (1|Subjects)	None	(1) Modality: Visual only(2) Word categories: Directional Words(3) Modality ∗ Word categories: Directional Words ∗ Visua only	648	0.007/0.297

## Appendix C. Subtitle-Level Analysis: Sanity Check

As a sanity check, those instructions that are necessary (i.e., when a new target agent is assigned) were isolated. The major eye-movement measures used here are total fixation counts, dwell time percentage, and subtitle skipping rates, as the three measures can reflect the general attention distribution of subtitles. The total fixation counts were calculated as the number of fixations on each subtitle, classified by modality and relevancy. The dwell time percentage was calculated as the dwell time on each subtitle divided by the presentation time of the subtitle aggregated by each modality and relevancy. Subtitle skipping rates were calculated as the probability of skipping each subtitle, classified by modality and relevancy, using the same method as the analysis of modality and relevancy in the previous section.

As shown in Figure A1, the overall patterns on each subtitle remain consistent in both the overall utterances and the essential utterances, but the subtitles are consistently processed in more detail when essential.
